# Further evaluation of inflammatory and non-inflammatory aspects of pain in rheumatoid arthritis patients

**DOI:** 10.1093/rap/rkad076

**Published:** 2023-09-20

**Authors:** Niels Jansen, Peter M ten Klooster, Harald E Vonkeman, Boudewijn van den Berg, Jan R Buitenweg

**Affiliations:** Biomedical Signals and Systems, Technical Medical Centre, University of Twente, Enschede, The Netherlands; Psychology, Health & Technology, Technical Medical Centre, University of Twente, Enschede, The Netherlands; Psychology, Health & Technology, Technical Medical Centre, University of Twente, Enschede, The Netherlands; Department of Rheumatology and Clinical Immunology, Medisch Spectrum Twente, Enschede, The Netherlands; Biomedical Signals and Systems, Technical Medical Centre, University of Twente, Enschede, The Netherlands; Biomedical Signals and Systems, Technical Medical Centre, University of Twente, Enschede, The Netherlands

**Keywords:** rheumatoid arthritis, non-inflammatory pain, quantitative sensory testing, DAS28, inflammation

## Abstract

**Objective:**

A high discrepancy between the number of tender and swollen joints (e.g. ΔTSJ ≥ 7) has previously been used as an indication for the presence of changes in central mechanisms in patients with moderate-to-high disease activity. In this study, we explored whether the ΔTSJ can also be used to obtain insights into the underlying pain mechanisms in patients with on average well-controlled disease activity.

**Methods:**

A 2 year retrospective analysis of routinely obtained 28-joint DAS (DAS28) components was performed on 45 patients with low inflammatory activity at the group level. All patients underwent pressure pain threshold (PPT) and electrical pain threshold (EPT) measurements and completed four self-report questionnaires [short-form 36 (SF-36v2); central sensitization inventory (CSI); generalized pain questionnaire (GPQ); and the pain catastrophizing scale (PCS)].

**Results:**

Patients with a ΔTSJ ≥ 3 at least once in the past 2 years showed significantly lower EPT and PPT values and higher levels of pain and disability on the SF-36v2 compared with the ΔTSJ < 3 group. Furthermore, GPQ scores were significantly higher in those with ΔTSJ ≥ 3, while CSI and PCS scores were similar.

**Conclusion:**

These findings suggest that in patients in the ΔTSJ ≥ 3 group, mechanisms other than inflammation (only) underlie the pain. Moreover, our findings suggest that among the multiple potential underlying psychological mechanisms, pain catastrophizing (as measured by the PCS) and psychological hypervigilance (as measured by the CSI) do not play an important role. These findings could be useful in the clinical management of the patient. Depending on the dominant mechanism underlying the (persistent) pain, patients might respond differently to treatment.

Key messages

- Using routinely obtained DAS28 measurements, indications on the dominant pain mechanism might be obtained.
- Depending on the dominant pain mechanism, patients with RA may respond differently to treatment.

## Introduction

The pharmacological treatment of RA has shown significant progress, with a growing number of patients achieving minimal disease activity according to the 28-joint DAS (DAS28) [1]. Despite these improvements, a substantial proportion of patients continue to experience moderate to severe levels of pain [2]. Interestingly, even in RA patients in DAS28 remission, ∼10–20% still report persistent pain in the absence of joint damage [3]. This suggests that the pain experienced by these patients is not driven solely by disease activity (i.e. peripheral and inflammation-driven mechanisms) but also involves changes in central pain regulatory mechanisms [4–7].

Understanding the dominant mechanism underlying pain in RA patients could be useful, because different subgroups of patients might respond differently to treatment approaches [8–10]. Therefore, it could be important to determine whether the pain is driven predominantly by disease activity or by changes in the central pain regulatory mechanisms. Recent evidence indicates that the number of swollen joints, rather than tender joints, is positively correlated with US-assessed joint inflammation [11–14]. Furthermore, negative correlations have been observed between tender joint counts and pressure pain thresholds (PPTs) measured at remote locations, suggesting alterations in central pain mechanisms [15–17]. These findings highlight the need for careful interpretation of DASs, such as the DAS28, in clinical practice, because the presence of mechanisms other than joint inflammation might lead to an overestimation of disease activity in some patients [18].

To assess the dominant underlying pain mechanism, a useful approach within daily clinical care is to monitor the difference between tender and swollen joint counts (ΔTSJ). Previous studies have provided indications for the feasibility of this approach, showing that the ΔTSJ can differentiate RA patients with an FM clinical phenotype (RA-FM) from a larger group of RA patients [19, 20]. Given that central mechanisms are believed predominantly to drive pain perception in RA-FM [4–7], this suggests that the ΔTSJ could be a valuable tool for identifying the dominant underlying pain mechanism in individual patients. In these previous studies, however, patients displaying high disease activity have been included primarily, leading to relatively high ΔTSJ values [19–21]. In current clinical practice, where most RA patients have well-controlled disease activity owing to treat-to-target principles [22], the numbers of swollen and tender joints, and consequently the ΔTSJ, are generally much lower [23]. This poses challenges to effective use of the ΔTSJ and joint counts in clinical practice for RA patients with well-controlled disease activity, because lower thresholds for the ΔTSJ might be more sensitive to random errors in joint counts.

In addition to the ΔTSJ, there are several instruments and tools available to gain insights into the involvement of central mechanisms underlying persistent pain. Quantitative sensory testing (QST) with standardized stimuli has been proposed as a valuable method for assessing central mechanisms [24]. By measuring pain thresholds at local and remote locations and comparing the results with a normative database, it is possible to evaluate the function of the peripheral and central nervous systems [25]. Self-report questionnaires, such as the central sensitization inventory (CSI) [26] and the generalized pain questionnaire (GPQ) [27], might also provide valuable information. The CSI, however, has been found to reflect more closely constructs related to psychological hypervigilance [28] rather than to manifestations caused by an increased responsiveness of central nociceptive neurons [29]. More recently, the GPQ [27] has been developed to identify the presence and intensity of generalized pain hypersensitivity, which is generally thought to be a manifestation of central sensitization [30]. The first findings appear promising, in that the GPQ has been found to distinguish RA patients from FM patients accurately. To date, however, no studies have investigated its convergent validity with QST measures.

In this cross-sectional study, we aimed to explore how routinely obtained DAS28 measurements can provide insights into the mechanisms underlying pain in RA patients with mostly well-controlled disease activity. We recruited a targeted sample of 46 RA patients and conducted QST measurements and standardized self-report questionnaires. Initially, we performed a retrospective analysis of DAS28 measurements over 2 years to investigate the numbers and variability of tender and swollen joint counts over time. Based on insights gained from this analysis, we stratified the patients into groups using historical ΔTSJ values, aiming to create approximately equal-sized groups with and without a ΔTSJ discrepancy. Finally, we compared the outcomes of the QST measurements and self-report questionnaires {CSI, GPQ and the pain catastrophizing scale (PCS) [31]} between these groups to gain a better understanding of the dominant underlying pain mechanisms.

## Methods

### Patients

Patients diagnosed with RA were included for QST and questionnaire measurements from September 2020 until August 2022. In total, 46 patients from the rheumatology department of the Medisch Spectrum Twente (MST) hospital in Enschede, The Netherlands were included. Owing to the exploratory nature of this study, no a priori power analysis was conducted. All QST and questionnaire measurements were performed at this outpatient location. All patients received written information before the study, signed an informed consent and were compensated for their time at the end of the study by provision of a voucher. Patients who were diagnosed with diabetes or PsA, who had an implanted stimulation device or who were pregnant were excluded. All these criteria were evaluated by asking patients for these conditions. The study was approved by the medical ethical committee united (MEC-U; reference number NL73282.100.20) and was conducted in accordance with the 1964 Helsinki Declaration and its later amendments.

Patients were recruited in two subsequent phases. Using the existing clinical dataset on outpatient RA patients, initially patients who had shown a ΔTSJ ≥ 4 at least once in the last 18 months were selected and contacted. After ∼20 patients had been included, additional patients were recruited from the remainder of the dataset (i.e. patients without a ΔTSJ ≥ 4 in the past 18 months). An attempt was made to match the patients from this second selection as closely as possible on age and sex to the patients already included. Importantly, the exact criterion on which we wanted to stratify the patients was not known before the analyses.

### Questionnaires

In total, five questionnaires were filled in by the patients at the end of the measurement session. A standardized case report form was developed to obtain general characteristics of the patient. This included a question to rate the level of pain perceived at that moment on an NRS ranging from 0 (no pain) to 10 (worst pain imaginable). The CSI [26] consists of two parts. Part A comprises 25 items that measure somatic and emotional complaints often associated with central sensitivity syndrome [32]. A score of ≥40 on part A of the questionnaire is indicative of the presence of central sensitization syndrome [33]. Part B of the questionnaire evaluates whether patients have been diagnosed with specific disorders being part of or related to central sensitivity syndrome [26]. The GPQ is a seven-item self-report instrument that assesses the presence and severity of various symptoms commonly associated with likely generalized pain hypersensitivity [27, 34]. A score of ≥11 is indicative of generalized pain hypersensitivity. The extent of pain catastrophizing was evaluated using the 13-item PCS, which quantifies the level of catastrophizing in both clinical and non-clinical populations [31]. The 36-item short form (SF-36v2) [35] was used to measure eight aspects of health-related quality of life, including physical functioning and bodily pain.orm

### Quantitative sensory testing

Measurements were performed by one of two trained experimenters. For photographs of the equipment used for the quantitative sensory testing, see Supplementary Fig. S1, available at *Rheumatology Advances in Practice* Online.

Pressure pain thresholds were evaluated bilaterally at the supraspinatus muscle and at the lateral epicondyle [36]. At these locations, pressure was increased by 50 kPa/s with a 1 cm^2^ probe using a battery-powered, hand-held algometer (Algometer Type II; SBMedic Electronics, Sweden). Patients were asked to indicate whenever the induced sensation became annoying for the first time, after which the examiner would stop applying pressure. Three subsequent measurements were performed and averaged into a single value.

Electrical pain thresholds (EPTs) were evaluated at the right upper arm at the intermediate part of the deltoid muscle [37] using patch electrodes (Red Dot 2560; 3M) with a surface area of 16 mm × 13.6 mm. Pulses of 100 Hz, with a width of 210 ms, were generated by a hand-held, constant-current stimulator (AmbuStim PT; University of Twente, Enschede, The Netherlands). When the patient pressed the button, the applied current ramped from 0 mA at 0.3 mA/s until a maximum current of 20 mA. The patient was asked to release the button (after which the current stopped immediately) whenever the sensation ascribed to application of the stimulus became annoying for the first time. Three subsequent measurements were performed, which were averaged into a single value.

### Retrospective analysis on the tender and swollen joints

After all patients had been included and measured, their DAS28-ESR measurements (tender joints, swollen joints, ESR, general health visual analog scale) over time were evaluated retrospectively. These routinely collected measurements were obtained from the electronic health records of the patient. For the analysis, a maximum of the four most recent DAS28 measurements in the past 2 years were considered. In most patients, DAS28-ESR measurements were not conducted on the day of the present study. As a best proxy for current disease activity and for disease activity classification [38], the most recent DAS28-ESR score of patients was used.

### Statistical testing

For continuous variables, group-level differences between the groups with and without a ΔTSJ discrepancy were evaluated using Student’s unpaired *t*-test [on log_10_-transformed data if a significant (*P* < 0.05) Shapiro–Wilk value was found] or a Mann–Whitney *U* test if the log_10_-transformation did not result in a normal distribution. For categorical variables, a χ^2^ test was used. Correlations were calculated using a two-tailed Pearson correlation coefficient. Statistical analyses were performed using MATLAB 2019b (The MathWorks Inc.).

## Results

### Retrospective analysis of routinely obtained DAS28-ESR outcomes

**Figure 1.**
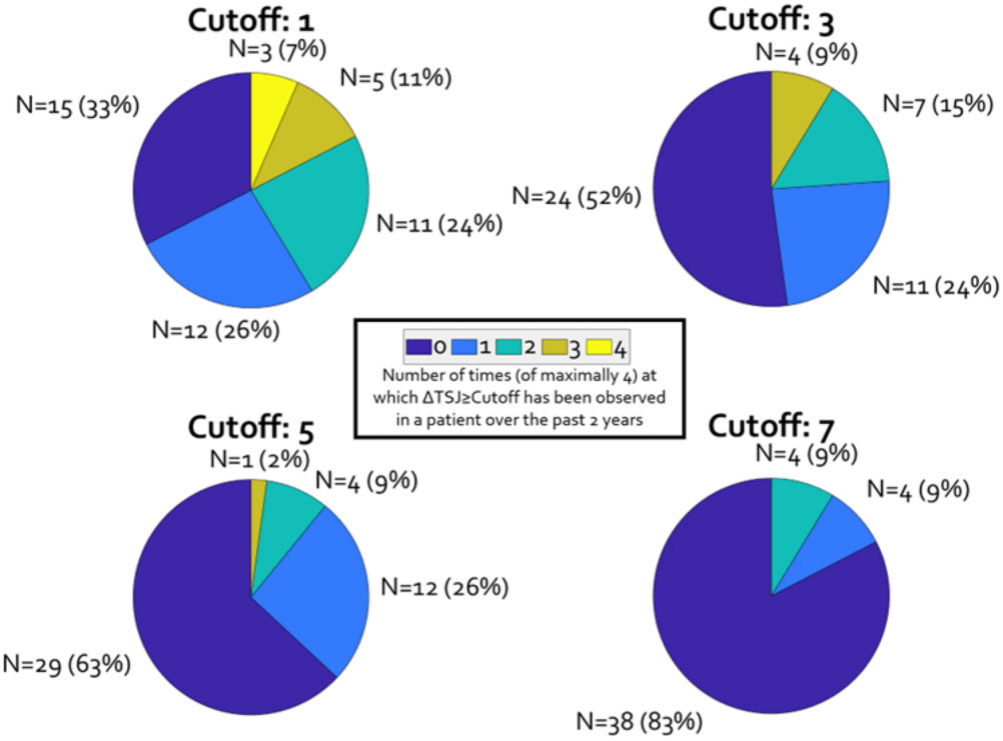
Overview of the historical difference between tender and swollen joint counts over a 2-year period. Per pie chart, the number of times a specific difference between tender and swollen joint counts (ΔTSJ) cut-off (of maximally four measurements) has been observed in the past 2 years since the measurement date is plotted. For instance, with a cut-off of three (top right), a total of 24 (52%) patients do not show such a discrepancy, while 11 (24%), 7 (15%) and 3 (9%) patients exhibit this discrepancy one, two or three times, respectively

For 33 of 46 patients, at least four DAS28-ESR measurements were conducted in the past 2 years. Of the remaining 13 patients, 11 had three available DAS28-ESR measurements; 1 patient (no. 27) had one available measurement and 1 patient (no. 38; excluded) did not have any DAS28-ESR measurements over the past 2 years. Of the 45 included patients, 31 patients had a ΔTSJ ≥ 1 at least once over the past 2 years. In total, 25, 22, 21 and 17 patients had at least one ΔTSJ of ≥2, ≥3, ≥4 and ≥5, respectively. For a more detailed overview of the observed ΔTSJ in the four most recent DAS28-ESR measurements, see Fig. 1. To create the groups roughly equal in size, a ΔTSJ ≥ 3 was chosen.

### Patient characteristics after grouping with a ΔTSJ ≥ 3

Using a ΔTSJ ≥ 3 for stratification resulted in a total of 23 patients without (ΔTSJ < 3) and 22 patients with a discrepancy (ΔTSJ ≥ 3) in their joint counts. For a full overview of baseline characteristics of the patients, see Table 1. Compared with the ΔTSJ < 3 group, the ΔTSJ ≥ 3 group reported a significantly higher number of tender joints and worse general health, but a significantly lower ESR. The number of swollen joints was not significantly different between the groups. The SF-36 bodily pain scale and the NRS confirmed that patients in the ΔTSJ ≥ 3 group experienced more pain than patients in the ΔTSJ < 3 group. The SF-36 also showed decreased physical functioning in the ΔTSJ ≥ 3 group. For the baseline characteristics explored for other possible ΔTSJ cut-offs (ΔTSJ ≥ 2, ΔTSJ ≥ 4 and ΔTSJ ≥ 5), see Supplementary Table S1, available at *Rheumatology Advances in Practice* Online.

**Table 1.** Patient baseline characteristics, divided using a difference between tender and swollen joint counts cut-off of three

Variable	ΔTSJ < 3	ΔTSJ ≥ 3	*P*-value
	(*n* = 23)	(*n* = 22)	
Age, mean (s.d.), years	55.0 (11.7)	61.0 (11.0)	0.08
Male, *n* (%)	12 (48)	5 (23)	0.16
BMI, mean (s.d.), kg/m^2^	25.6 (3.1)	27.6 (3.7)	0.06
Smoking in last 24 h, mean (s.d.)	1.5 (3.4)	3.5 (6.8)	0.23
Alcohol in last 24 h, mean (s.d.)	0.17 (0.7)	0.70 (2.6)	0.35
Sport in last 24 h, mean (s.d.), h	1.3 (3.1)	1.1 (2.0)	0.81
Sleep in last 24 h, mean (s.d.), h	7.4 (1.1)	6.8 (1.6)	0.28
Right-handedness, *n* (%)	19 (95)	17 (77)	0.65
Disease duration, median (IQR), years	11.1 (7.5)	11.5 (8.2)	0.86
Erosive, *n* (%)^a^	6 (35)	8 (40)	0.84
RF positive, *n* (%)^b^	22 (96)	12 (63)	0.38
DAS28, mean (s.d. [min–max])	2.3 (1.0; [0.5–4.6])	2.9 (1.4; [0.9–5.8])	0.09
Classification, *n* [remission; low; middle; high]	[11; 3; 4; 5]	[7; 6; 4; 5]	0.86
Tender joints, *n*	0.5 (0.9; [0–3])	4.9 (6.2; [0–24])	<0.01
Swollen joints, *n*	0.6 (1.4; [0–6])	1.5 (3.3; [0–11])	0.22
ESR	13.6 (13.2; [2–54])	7.4 (11.7; [2–51])	0.02
General health	32.8 (24.6; [0–95])	55.9 (24.4; [0–95])	<0.01
Painkillers, *n* (%)			
NSAIDs	15 (65)	15 (68)	0.92
Opioids	2 (9)	6 (27)	0.17
Medication use, *n* (%)			
csDMARD	19 (83)	15 (68)	0.67
bDMARD	12 (52)	10 (45)	0.79
tsDMARD	0 (0)	2 (9)	0.16
Dutch SF-36, mean (s.d.)			
Physical functioning	66.3 (22.6)	52.5 (21.1)	0.04
Role physical	47.8 (15.0)	42.3 (17.6)	0.26
Bodily pain	61.1 (17.3)	47.5 (23.9)	0.03
General health	48.0 (18.9)	49.6 (20.1)	0.82
Vitality	53.9 (14.0)	49.5 (16.5)	0.34
Social functioning	79.3 (19.1)	72.3 (20.6)	0.30
Role emotional	68.1 (15.0)	59.4 (20.9)	0.17
Mental health	72.0 (10.2)	73.8 (13.3)	0.61
NRS pain (0–10), mean (s.d.)	3.4 (2.1)	5.0 (2.6)	0.03

a Status is unknown in eight patients. Percentages are calculated using only the data of patients where the status is known.

b Status is unknown in three patients. Percentages are calculated using only the data of patients where the status is known.

bDMARD: biological DMARD; csDMARD: conventional synthetic DMARD; DAS28: DAS at the last measurement at which all components were known; NRS: numerical rating score; tsDMARD: targeted synthetic DMARD; ΔTSJ: difference between tender and swollen joint counts.

For an overview of responses to the questionnaires and pain threshold measurements, see Table 2. Lower pain thresholds were observed in the ΔTSJ ≥ 3 group response to the electrical and pressure stimuli at the left and right lateral epicondyle. The PPTs measured at the left and right supraspinatus muscle were not significantly different between the groups. Patients in the ΔTSJ ≥ 3 group had significantly higher scores on the GPQ, whereas no significant differences were found between the groups for the scores of the CSI or PCS. For responses to the questionnaires and pain threshold measurements at other possible ΔTSJ cut-offs (ΔTSJ ≥ 2, ΔTSJ ≥ 4 and ΔTSJ ≥ 5), see Supplementary Table S1, available at *Rheumatology Advances in Practice* Online.

**Table 2.** Responses to the questionnaires and pain threshold measurements

Variable	ΔTSJ < 3	ΔTSJ ≥ 3	*P*-value
	(*n* = 23)	(*n* = 22)	
Generalized pain questionnaire	5.1 (3.8)	9.4 (5.9)	<0.01
Central sensitization inventory			
Part A	30.4 (12.1)	36.0 (16.4)	0.20
Part B	0.78 (0.9)	1.14 (1.9)	0.43
Pain catastrophizing scale	9.7 (8.1)	11.1 (10.0)	0.60
Pressure pain threshold, kPa			
Epicondyle (L)	443 (169)	302 (156)	<0.01
Epicondyle (R)	463 (161)	311 (140)	<0.01
Supraspinatus (L)	379 (146)	322 (182)	0.07
Supraspinatus (R)	378 (157)	328 (169)	0.16
Electrical pain threshold, mA	10.9 (4.9)	7.7 (3.7)	<0.01

The responses to the questionnaires (generalized pain questionnaire, central sensitization inventory and pain catastrophizing scale) and the pain threshold measurements (pressure pain threshold and electrical pain threshold) are shown for the patients who displayed a ΔTSJ≥3 at least once over the last 2 years or at the last four measurements (ΔTSJ≥3) and for those who did not display this (ΔTSJ<3).

L: left; R: right; ΔTSJ: difference between tender and swollen joint counts.

### Correlations between questionnaires and pain sensitivity measurements

In the total sample (*n* = 45), significant moderate to strong correlations were found between the CSI and the PCS (*r* = 0.46; *P* < 0.001) and between the GPQ and the CSI (*r* = 0.70; *P* < 0.001). The GPQ was correlated significantly, but more weakly than the CSI, with the PCS (*r* = 0.30; *P* < 0.05). The GPQ was correlated significantly with the PPTs measured at the left (*r* = −0.35; *P* < 0.05) and right (*r* = −0.38; *P* < 0.05) lateral epicondyle. Between the GPQ and the PPT measured at the left (*r* = −0.39) and right (*r* = −0.29) supraspinatus muscle and the EPT (*r* = −0.30), near-significant (*P* < 0.075) correlations were found. In the ΔTSJ < 3 group (*n* = 23), a significant correlation was found only between the CSI and the GPQ questionnaires (*r* = 0.67; *P* < 0.01). In the ΔTSJ ≥ 3 group (*n* = 22), statistically significant correlations were also found between the CSI and the PCS (*r* = 0.52; *P* < 0.05). Additionally, in this group the GPQ was significantly correlated with the PPT measured at the left (*r* = −0.52; *P* < 0.05) and right (*r* = −0.52; *P* < 0.05) lateral epicondyle and on the left supraspinatus muscle (*r* = −0.45; *P* < 0.05). Lastly, a near-significant correlation (*P* < 0.075) between the GPQ and EPT was observed (*r* = −0.45). For a full overview of all correlations, see Table 3.

**Table 3.** Correlations between outcome measures

	GPQ	CSI	PCS	PPT				EPT
				Epicondyle		Supraspinatus		
				L	R	L	R	
All patients (*n*=45)								
GPQ	—	0.70***	0.30*	−0.35*	−0.38*	−0.39^†^	−0.29^†^	−0.30^†^
CSI	0.70***	—	0.46**	−0.18	−0.11	−0.23	−0.11	−0.14
PCS	0.30*	0.46**	—	−0.04	−0.09	−0.16	−0.12	−0.07
ΔTSJ<3 group only (*n*=23)								
GPQ	—	0.67**	0.25	0.26	0.20	−0.18	−0.06	0.15
CSI	0.67**	—	0.35	0.17	0.22	−0.38	−0.21	0.03
PCS	0.25	0.35	—	0.24	0.09	−0.12	−0.02	0.20
ΔTSJ≥3 group only (*n*=22)								
GPQ	—	0.72***	0.33	−0.52*	−0.52*	−0.45*	−0.36	−0.45^†^
CSI	0.72***	—	0.52*	−0.32	−0.23	−0.10	0.00	−0.17
PCS	0.34	0.52*	—	−0.23	−0.19	−0.17	−0.22	−0.31

Correlations between the questionnaires (generalized pain questionnaire, central sensitization inventory and pain catastrophizing scale) and the pain threshold measurements (pressure pain threshold and electrical pain threshold) are shown, computed on the complete patient group (All patients), on the patients who displayed a ΔTSJ≥3 at least once over the last 2 years or at the last four measurements (ΔTSJ≥3) and on those who did not display this (ΔTSJ<3).

* *P*<0.05,

** *P*<0.01,

*** *P*<0.001,

† *P*<0.075.

CSI: central sensitization inventory; EPT: electrical pain threshold; GPQ: generalized pain questionnaire; L: left; PCS: pain catastrophizing scale; PPT: pressure pain threshold; R: right; ΔTSJ: difference between tender and swollen joint counts.

## Discussion

The aim of this study was to explore how routinely obtained DAS28 measurements can be used to gain insight into the mechanisms underlying pain in RA patients. Overall, the findings suggest that by using a single historical (2 year) cut-off in ΔTSJ of ≥3 in patients with well-controlled disease activity, patients can be identified who display lower EPT and PPT values and higher GPQ scores and who also report higher levels of pain and disability. These findings suggest that mechanisms other than joint inflammation alone underlie the pain in these patients. Moreover, finding no differences in CSI and PCS scores suggests that commonly believed psychological processes of pain catastrophizing and psychological hypervigilance do not play an important role.

Previous studies examining tender and swollen joint counts, in addition to ΔTSJ, have focused primarily on patients with high inflammatory activity, as indicated by an elevated ESR and/or a high baseline number of swollen joints [9, 15–17, 19, 20] (see Supplementary Table S2, available at *Rheumatology Advances in Practice* Online). However, we observed lower levels of inflammation and swollen joints overall, which aligns better with the well-controlled patients commonly encountered in current clinical practice, in which treat-to-target principles are adopted [23].

In this retrospective analysis of DAS28 outcomes collected during routine clinical practice over a 2 year period, we found that eight (18%) patients exhibited a ΔTSJ ≥ 7 at least once during the last 2 years or within the last four visits. This contrasts with previous studies, where such discrepancies were typically observed in only one measurement [19, 20]. Notably, we intentionally included patients with a ΔTSJ ≥ 4 (at least once in the last 18 months) during the initial recruitment phase of our study. Consequently, our sample might overrepresent patients with a higher likelihood of experiencing discrepancies. Our findings indicate that patients with well-controlled disease activity have significantly lower ΔTSJ values compared with patients who have high disease activity, as observed in previous studies [9, 15–17, 20].

We expected initially to observe random or natural variability in both swollen and tender joint counts over time, which would also lead to discrepancies between measurements. Variations in the severity of inflammation, such as those caused by life events or changes in medication, can result in fluctuations in the number of swollen and tender joints over time. Additionally, the way in which joint assessments are conducted could influence the outcomes. For instance, the number of swollen joints can vary depending on visual inspection performed by different clinicians or nurses, and the assessment of painful joints relies on the subjective experience of the patient following gentle pressure applied by the clinician or nurse (±4 kg/cm^2^). Our retrospective analysis did reveal such minor variability, as evidenced by 31 (67%) patients exhibiting at least one discrepancy (ΔTSJ ≥ 1) over time.

Finally, we observed considerable changes in ΔTSJ between visits, surpassing what could be expected based on the minor variability described earlier. For instance, the ΔTSJ of a patient could be (near) zero during one visit and then increase to nine during the next visit. This indicates that the ΔTSJ of a patient is not consistently high, suggesting caution when interpreting a single measurement of ΔTSJ.

Earlier studies used a ΔTSJ ≥ 7 as a grouping variable to identify RA-FM patients [19, 20] or a swollen-to-tender ratio to predict treatment response [21]. Our retrospective analysis of joint counts revealed that only a small number of patients had a ΔTSJ ≥ 7 over 2 years. Therefore, using a cut-off of ΔTSJ ≥ 7 is not feasible in patients with well-controlled disease activity. Additionally, many measurements showed zero tender joints (see Fig. 1), making the use of a ratio score impossible. We therefore took a pragmatic grouping approach, in which the history of the patient was also included, with the primary aim being to obtain groups approximately equal in size.

Using a ΔTSJ ≥ 3 (or ΔTSJ ≥ 4 or ΔTSJ ≥ 5; see Supplementary Table S1, available at *Rheumatology Advances in Practice* Online) resulted in lower EPTs and PPTs at the left and right lateral epicondyle in the ΔTSJ ≥ 3 group compared with the ΔTSJ < 3 group. PPT measurements at the supraspinatus muscle showed no significant differences between the groups, although a near-significant difference was observed on the left side, which was, for most patients, the non-dominant, more sensitive side [39]. The PPT measurements at the lateral epicondyle, located near the elbow joint, probably involved peripheral mechanisms. Such peripheral contributions are unlikely with the EPT measurements performed at the right deltoid muscle and the PPT measurements performed at the supraspinatus muscle, because these locations are both away from joints. As such, the significantly lower EPT in ΔTSJ ≥ 3 patients is indicative of the presence of central neural mechanisms acting on a segmental level. In contrast, the non-significant differences in PPTs at the supraspinatus muscle do not indicate central mechanisms acting at an extra-segmental level.

Furthermore, it was found that when using a ΔTSJ ≥ 3 (or ΔTSJ ≥ 4 or ΔTSJ ≥ 5; see Supplementary Table S1, available at *Rheumatology Advances in Practice* Online), higher physical disability and pain levels in the ΔTSJ ≥ 3 group could be observed compared with the ΔTSJ < 3 group. These findings, obtained by using a substantially lower ΔTSJ cut-off and by including historical measurements, are similar to the earlier studies, whereby a ΔTSJ cut-off of ≥7 was used to identify patients with characteristics of RA-FM [19, 20]. Finding an RA-FM clinical phenotype in the patients in the ΔTSJ ≥ 3 group is unsurprising after having established the presence of central mechanisms, because divergent central pain regulatory mechanisms are commonly believed to underlie the symptoms of FM [4–7]. It has been suggested that FM progresses from chronic regional musculoskeletal pain to widespread pain [40]. As such, the patients in the ΔTSJ ≥ 3 group being in varying developmental stages might explain why evidence was found only for the presence of central mechanisms acting at a segmental level, but not for central mechanisms acting at an extra-segmental level.

Various pro- and anti-nociceptive central mechanisms exist [41], which can affect both the ascending nociceptive signal and the emotional–affective appraisal of pain perception [42]. These mechanisms can have widespread effects or act at a segmental or extra-segmental level [25]. From the results of the assessments in the present study, we are, however, able to indicate some commonly believed mechanisms that are less likely to be involved. Here, the ΔTSJ ≥ 3 group exhibited higher GPQ scores compared with the ΔTSJ < 3 group, but no significant differences in CSI or PCS scores. The CSI is frequently used to evaluate the presence and severity of central sensitization. Recently, however, it has been hypothesized that the CSI more closely reflects psychological hypervigilance rather than an increased responsiveness of nociceptive neurons [28], because it shows weak or no associations with experimental nociceptive sensitivity, yet strong correlations with psychological constructs such as pain catastrophizing, as measured by the PCS [28]. As such, not finding group-level differences with these questionnaires suggests that pain catastrophizing and psychological hypervigilance are not involved.

Consistent with previous research, we found no significant correlations between the CSI and EPT or PPT measurements, but strong correlations between the CSI and PCS. Notably, the EPT and PPT measurements were correlated with the GPQ, particularly in the ΔTSJ ≥ 3 group. These results provide initial support for the convergent validity of the GPQ as a tool to assess generalized pain hypersensitivity [27].

The results of this study provide several directions for further research. First, it could be investigated which specific central mechanisms are involved in this ΔTSJ ≥ 3 patient group. It is worth noting that such insights, although useful from a scientific point of view, might provide limited added value for clinical practice, because for that purpose it might suffice to know whether the dominant underlying pain mechanism is not driven solely by disease activity. Second, the present cross-sectional study (better) justifies and provides insights for designing a prospective study that could investigate the relationship between the pain threshold measurements and questionnaires with the ΔTSJ over time. Such a prospective study might also provide insights into the developmental trajectories of the underlying pain mechanisms in patients.

### Conclusion

By grouping RA patients using a ΔTSJ based on the most recent DAS28-ESR measurements during a 2 year period rather than a single measurement, at a group level patients could be identified with lower EPTs and PPTs and higher GPQ scores, while also displaying higher levels of pain and disability. These findings suggest that in these patients, mechanisms other than solely inflammatory mechanisms underlie the (persistent) pain. Our results also suggest that among the multiple potential underlying mechanisms, pain catastrophizing and psychological hypervigilance do not play an important role. These findings could be useful in the clinical management of the patient because, depending on the dominant mechanism underlying the (persistent) pain, patients might respond differently to treatment.

## Supplementary data

Supplementary data are available at *Rheumatology Advances in Practice* Online.

## Supplementary Material

rkad076_Supplementary_Data

## Data Availability

The data underlying this article will be shared on reasonable request to the corresponding author.
