# Supplementary material for: Further evaluation of inflammatory and non-inflammatory aspects of pain in rheumatoid arthritis patients
Source: Rheumatol Adv Pract. 2023 Sep 20;7(3):rkad076. doi: 10.1093/rap/rkad076 (PMC10560383; doi:10.1093/rap/rkad076)
Supplement: rkad076_Supplementary_Data [file rkad076_supplementary_data.docx]

| 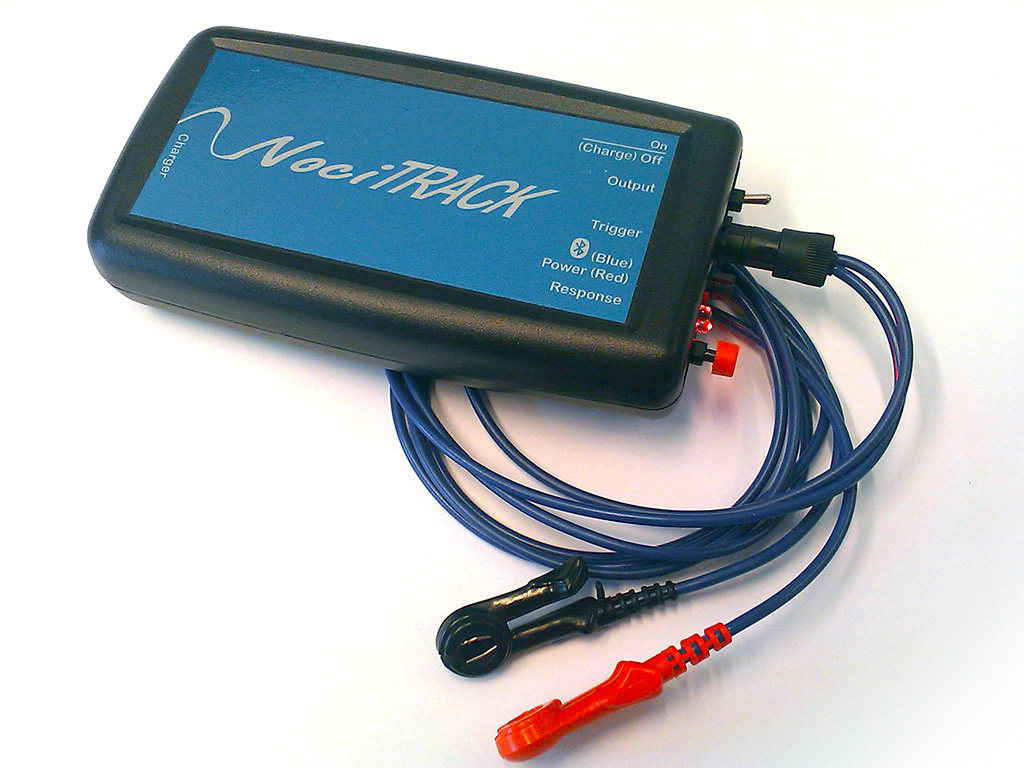 | 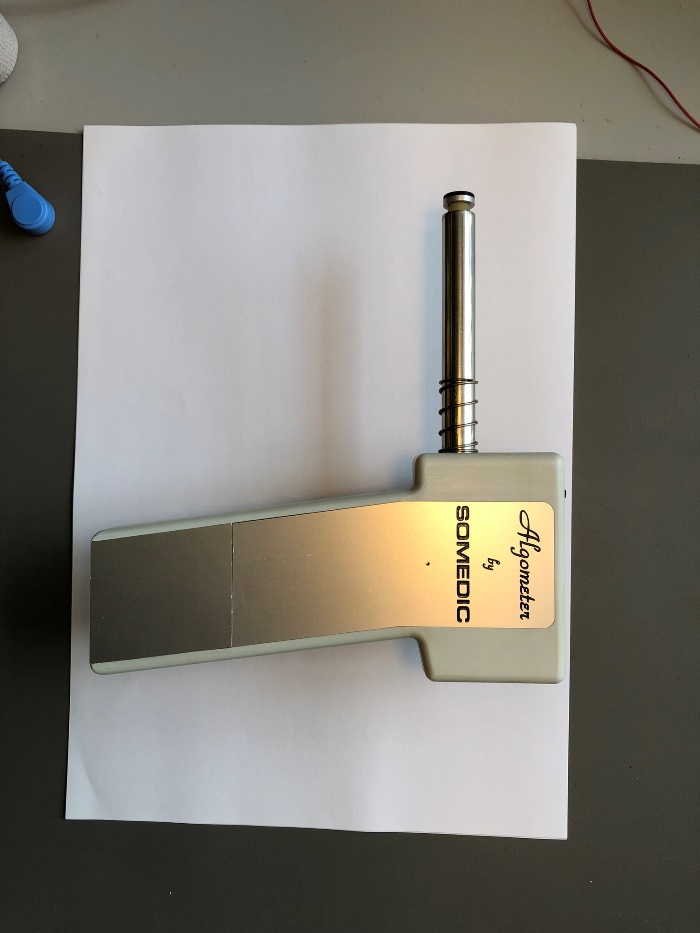 |
| --- | --- |
| **A** | **B** |
| **Supplementary Figure S1: Photos of the equipment used during this study.** (A) The AmbuStim PT (University of Twente, Enschede, The Netherlands) has a constant-current generator. When the patient presses the red button on the stimulator, the current ramps from 0 to maximally 20 mA with 0.3 mA/s. (B) The Algometer (Type II, SBMedic Electronics, Sweden). In this study a 1 cm^2^ probe was used and the pressure was increased with 50 kPa/s. | |

| **Supplementary Table S1.** In the table, characteristics as well as responses to the questionnaires (CSI, GPQ and PCS) and the pain threshold measurements (PPT and EPT) of the patients are shown which are grouped using an increasingly higher ΔTSJ cutoff value. | | | | | | | | | | | | | | | | | | |
| --- | --- | --- | --- | --- | --- | --- | --- | --- | --- | --- | --- | --- | --- | --- | --- | --- | --- | --- |
|  | **All Patients** | | **ΔTSJ<2** | | **ΔTSJ≥2** | | **ΔTSJ<3** | | **ΔTSJ≥3** | | **ΔTSJ<4** | | **ΔTSJ≥4** | | **ΔTSJ<5** | | **ΔTSJ≥5** | |
|  | **N / Mean** | **SD / %** | **N / Mean** | **SD / %** | **N / Mean** | **SD / %** | **N / Mean** | **SD / %** | **N / Mean** | **SD / %** | **N / Mean** | **SD / %** | **N / Mean** | **SD / %** | **N / Mean** | **SD / %** | **N / Mean** | **SD / %** |
| **N** | 45 | 100 | 20 | 44 | 25 | 56 | 23 | 51 | 22 | 49 | 24 | 53 | 21 | 47 | 28 | 62 | 17 | 38 |
| **Age (years), mean (SD)** | 58 | 11.6 | 54.1 | 11.8 | 61.1* | 10.7 | 55.0 | 11.7 | 61.0 | 11.0 | 56.0 | 12.3 | 60.3 | 10.7 | 55.8 | 12.0 | 61.6 | 10.4 |
| **Male, n (%)** | 17 | 38 | 10 | 50 | 7 | 38 | 12 | 48 | 5 | 23 | 13 | 54 | 4 | 19 | 13 | 46 | 4 | 24 |
| **BMI (kg/m^2^), mean (SD)** | 26.6 | 3.5 | 25.4 | 3.2 | 27.5* | 3.6 | 25.6 | 3.1 | 27.6 | 3.7 | 25.8 | 3.2 | 27.5 | 3.8 | 26.4 | 3.8 | 26.8 | 3.1 |
| **Smoking last 24h, mean (SD)** | 2.5 | 5.4 | 1.3 | 3.0 | 3.4 | 6.6 | 1.5 | 3.4 | 3.5 | 6.8 | 1.5 | 3.3 | 3.6 | 7.0 | 1.3 | 3.1 | 4.4 | 7.5 |
| **Alcohol last 24h, mean (SD)** | 0.4 | 1.9 | 0.2 | 0.7 | 0.7 | 2.4 | 0.2 | 0.7 | 0.7 | 2.6 | 0.2 | 0.6 | 0.7 | 2.7 | 0.1 | 0.6 | 0.9 | 2.9 |
| **Sport last 24h (h), mean (SD)** | 1.2 | 2.6 | 1.4 | 3.3 | 1.0 | 1.9 | 1.3 | 3.1 | 1.1 | 2.0 | 1.2 | 3.1 | 1.1 | 2.0 | 1.2 | 2.8 | 1.2 | 2.2 |
| **Sleep last 24h (h), mean (SD)** | 7.1 | 1.4 | 7.3 | 1.2 | 6.9 | 1.5 | 7.4 | 1.1 | 6.8 | 1.6 | 7.3 | 1.2 | 6.9 | 1.5 | 7.3 | 1.1 | 6.9 | 1.7 |
| **Right-handedness, n (%)** | 36 | 86 | 16 | 94 | 20 | 80 | 19 | 95 | 17 | 77 | 20 | 95 | 16 | 76 | 24 | 96 | 12 | 71 |
| **Disease duration (years), median (IQR)** | 11.3 | 7.8 | 11.3 | 7.6 | 11.4 | 8.1 | 11.1 | 7.5 | 11.5 | 8.2 | 11.3 | 7.3 | 11.4 | 8.4 | 10.9 | 7.0 | 12.0 | 9.0 |
| **Erosive, n (%) ^1^** | 14 | 38 | 5 | 36 | 9 | 39 | 6 | 35 | 8 | 40 | 6 | 33 | 8 | 42 | 9 | 41 | 5 | 33 |
| **RF positive, n (%) ^2^** | 34 | 81 | 19 | 95 | 15 | 68 | 22 | 96 | 12 | 63 | 23 | 96 | 11 | 61 | 24 | 89 | 10 | 67 |
| **DAS28, mean (SD)** | 2.6 | 1.2 | 2.3 | 1.1 | 2.9 | 1.3 | 2.3 | 1.1 | 2.9 | 1.4 | 2.3 | 1.0 | 2.9 | 1.4 | 2.3 | 1.1 | 3.1* | 1.4 |
| Classification (N)  [Remission; Low; Middle; High] | [18; 9; 8; 10] | | [10; 2; 4; 4] | | [8; 7; 4; 6] | | [11; 3; 4; 5] | | [7; 6; 4; 5] | | [12; 3; 4; 5] | | [6; 6; 4; 5] | | [14; 4; 4; 6] | | [4; 5; 4; 4] | |
| Tender joints (N) | 2.7 | 4.9 | 0.5 | 0.8 | 4.4** | 6.0 | 0.5 | 0.9 | 4.9** | 6.2 | 0.6 | 1.0 | 5.0** | 6.4 | 0.9 | 1.3 | 5.6** | 6.9 |
| Swollen joints (N) | 1.0 | 2.5 | 0.6 | 1.5 | 1.4 | 3.1 | 0.6 | 1.4 | 1.5 | 3.3 | 0.5 | 1.4 | 1.6 | 3.3 | 0.5 | 1.3 | 1.9 | 3.7 |
| ESR | 10.7 | 12.8 | 12.5 | 12.8 | 9.2 | 12.8 | 13.6 | 13.2 | 7.4* | 11.7 | 13.1 | 13.1 | 7.7* | 11.9 | 12.2 | 13.0 | 8.4 | 12.5 |
| General Health | 44.0 | 26.9 | 33.4 | 25.8 | 52.7* | 25.1 | 32.8 | 24.6 | 55.9** | 24.4 | 34.0 | 24.7 | 55.7** | 25.0 | 36.9 | 25.7 | 56.2* | 25.0 |
| **Painkillers, n (%)** |  |  |  |  |  |  |  |  |  |  |  |  |  |  |  |  |  |  |
| NSAIDs | 30 | 67 | 13 | 65 | 17 | 68 | 15 | 65 | 15 | 68 | 15 | 63 | 15 | 71 | 18 | 64 | 12 | 71 |
| Opioids | 8 | 18 | 2 | 10 | 6 | 24 | 2 | 9 | 6 | 27 | 2 | 8 | 6 | 29 | 3 | 11 | 5 | 29 |
| **Medication use, n (%)** |  |  |  |  |  |  |  |  |  |  |  |  |  |  |  |  |  |  |
| csDMARD | 34 | 76 | 17 | 85 | 17 | 68 | 19 | 83 | 15 | 68 | 20 | 83 | 14 | 67 | 24 | 86 | 10 | 54 |
| bDMARD | 22 | 49 | 11 | 55 | 11 | 44 | 12 | 52 | 10 | 45 | 12 | 50 | 10 | 48 | 15 | 54 | 7 | 41 |
| tsDMARD | 2 | 0.4 | 0 | 0 | 2 | 8 | 0 | 0 | 2 | 9 | 0 | 0 | 2 | 10 | 0 | 0 | 2 | 12 |
| **Dutch SF-36, mean (SD)** |  |  |  |  |  |  |  |  |  |  |  |  |  |  |  |  |  |  |
| Physical Functioning | 59.6 | 22.7 | 65.5 | 22.4 | 54.8 | 22.3 | 66.3 | 22.6 | 52.5* | 21.1 | 65.6 | 22.4 | 52.6^#^ | 21.6 | 65.7 | 22.3 | 49.4* | 20.1 |
| Role Physical | 45.1 | 16.4 | 46.8 | 15.3 | 43.8 | 17.5 | 47.8 | 15.1 | 42.3 | 17.6 | 47.5 | 14.8 | 42.4 | 18.1 | 48.8 | 15.1 | 39.1 | 17.2 |
| Bodily Pain | 54.4 | 21.7 | 59.6 | 17.8 | 50.3 | 23.9 | 61.1 | 17.3 | 47.5* | 23.9 | 61.4 | 17.0 | 46.5* | 24.0 | 61.8 | 17.9 | 42.4** | 22.3 |
| General Health | 48.8 | 19.3 | 48.3 | 18.9 | 49.2 | 20.0 | 48.0 | 18.9 | 49.5 | 20.1 | 48.3 | 18.6 | 49.3 | 20.6 | 48.2 | 19.1 | 49.7 | 20.2 |
| Vitality | 51.8 | 15.2 | 52.0 | 13.6 | 51.6 | 16.7 | 53.9 | 14.0 | 49.5 | 16.5 | 53.8 | 13.7 | 49.5 | 16.9 | 54.8 | 14.4 | 46.8 | 15.7 |
| Social Functioning | 76.1 | 19.9 | 78.1 | 19.0 | 74.5 | 20.9 | 79.3 | 19.1 | 72.7 | 20.6 | 78.6 | 19.0 | 73.2 | 21.0 | 81.3 | 18.8 | 67.6* | 19.3 |
| Role Emotional | 63.9 | 18.4 | 66.3 | 15.4 | 61.9 | 20.7 | 68.1 | 15.0 | 59.4 | 20.9 | 66.9 | 15.8 | 60.3 | 20.9 | 67.9 | 15.1 | 57.3 | 21.9 |
| Mental Health | 72.9 | 11.7 | 71.2 | 10.6 | 74.2 | 12.6 | 72.0 | 10.2 | 73.8 | 13.3 | 71.3 | 10.5 | 74.7 | 13.0 | 72.9 | 11.2 | 72.9 | 12.9 |
| **NRS pain (0-10), mean (SD)** | 4.1 | 2.5 | 3.5 | 2.0 | 4.7 | 2.7 | 3.4 | 2.1 | 5.0* | 2.6 | 3.4 | 2.1 | 5.0* | 2.7 | 3.6 | 2.2 | 5.1* | 2.7 |
|  |  |  |  |  |  |  |  |  |  |  |  |  |  |  |  |  |  |  |
| ***Generalized Pain Questionnaire*** | 7.3 | 5.3 | 5.9 | 3.5 | 8.3 | 6.2 | 5.1 | 3.8 | 9.4** | 5.9 | 5.2 | 3.7 | 9.5** | 6.0 | 5.2 | 4.1 | 10.5** | 5.5 |
| ***Central Sensitization Inventory*** |  |  |  |  |  |  |  |  |  |  |  |  |  |  |  |  |  |  |
| Part A | 33.1 | 14.5 | 30.8 | 12.3 | 35.0 | 16.0 | 30.4 | 12.1 | 36.0 | 16.4 | 30.7 | 11.9 | 36.0 | 16.8 | 30.4 | 13.1 | 37.7 | 15.8 |
| Part B | 1.0 | 1.5 | 0.7 | 0.7 | 1.2 | 1.9 | 0.8 | 0.9 | 1.1 | 1.9 | 0.8 | 0.8 | 1.2 | 2.0 | 0.8 | 0.8 | 1.3 | 2.1 |
| ***Pain Catastrophizing Scale*** | 10.4 | 9.0 | 9.5 | 8.6 | 11.1 | 9.5 | 9.7 | 8.1 | 11.1 | 10.0 | 9.4 | 8.1 | 11.6 | 10.1 | 9.8 | 8.3 | 11.5 | 10.4 |
| ***Pressure Pain Threshold [kPa]*** |  |  |  |  |  |  |  |  |  |  |  |  |  |  |  |  |  |  |
| Epicondyle (L) | 372 | 176 | 462 | 169 | 304** | 150 | 443 | 169 | 302** | 156 | 442 | 168 | 302** | 156 | 409 | 175 | 317 | 166 |
| Epicondyle (R) | 388 | 168 | 479 | 159 | 319** | 142 | 463 | 161 | 311** | 140 | 463 | 161 | 311** | 140 | 428 | 177 | 328 | 137 |
| Supraspinatus (L) | 349 | 167 | 387 | 143 | 324 | 178 | 379 | 146 | 322 | 182 | 379 | 146 | 322 | 182 | 356 | 152 | 339 | 188 |
| Supraspinatus (R) | 353 | 163 | 385 | 153 | 329 | 169 | 377 | 157 | 328 | 169 | 377 | 157 | 328 | 169 | 357 | 158 | 346 | 175 |
| ***Electrical Pain Threshold [mA]*** | 9.4 | 4.6 | 11.4 | 4.6 | 7.6** | 3.9 | 10.9 | 4.9 | 7.7* | 3.7 | 11.1 | 4.9 | 7.2** | 3.0 | 10.6 | 4.9 | 7.1* | 3.1 |
| BMI = Body Mass Index, RF = Rheumatoid factor, DAS28 = Disease activity score at the last measurement at which all components were known, DMARD = disease-modifying anti-rheumatic drug, csDMARD = conventional synthetic DMARD, bDMARD = biological DMARD, tsDMARD = targeted synthetic DMARD, NRS = Numerical Rating Score. **^1^** Status is unknown in 8 patients. Percentages are calculated using only the data of patients where the status is known. **^2^** Status is unknown in 3 patients. Percentages are calculated using only the data of patients where the status is known. | | | | | | | | | | | | | | | | | | |

| Supplementary Table S2. Overview of studies which report on ΔTSJ. | | | | | | | |
| --- | --- | --- | --- | --- | --- | --- | --- |
| Paper | **Criterium** | **Female (%)** | **DAS28** | **GH** | **ESR (mm/hr)** | **Swollen (#)** | **Tender (#)** |
| This study | ΔTSJ<3 | 52 | 2.3 [0.5-4.6] | 33 [0-95] | 13.6 [2-54] | 0.6 [0-6] | 0.5 [0-3] |
|  | ΔTSJ≥3 | 77 | 2.9 [0.9-5.8] | 56 [0-95] | 7.4 [2-51] | 1.5 [0-11] | 4.9 [0-24] |
|  |  |  |  |  |  |  |  |
| Joharatnam et al. (2015) | FM (No) | 77 | 4.4 [3.8-4.9] | 42 [24-55] | ? | 1 [1-2] | 6 [4-9] |
|  | FM (Yes) | 75 | 4.8 [4.4-5.3] | 70 [55-78] | ? | 1 [0-2] | 11 [7-18] |
|  |  |  |  |  |  |  |  |
| Pollard, Choy, and Scott (2005) | ΔTSJ<7 | ? | 4.0 [3.7-4.3] | 37 [31-42] | 33 [22-43] | 4 [3-4] | 4 [3-5] |
|  | ΔTSJ≥7 | ? | 5.7 [5.3-6.1] | 61 [53-68] | 28 [24-32] | 3 [2-4] | 16 [14-18] |
|  |  |  |  |  |  |  |  |
| Kapoor, Hider, Brownfield, Mattey, and Packham (2011) | ΔTSJ<7 | 70 | 3.8 [3.0-4.8] | 39 [20-55] | 16 [10-30] | 3 [1-6] | 3 [1-6] |
|  | ΔTSJ≥7 | 77 | 5.4 [4.8-6.5] | 55 [34-75] | 34 [16-46] | 3 [1-7] | 14 [11-18] |
|  |  |  |  |  |  |  |  |
| Kristensen et al. (2014) | STR^2^ < 0.5 | 77 | 5.2 [4.2-6.1] | 69 [52-80] | 7.8 [1.7-22.2] ^1^ | 2 [1-5] | 10 [6-17] |
|  | STR^2^ 0.5 - 1.0 | 80 | 5.8 [4.9-6.6] | 68 [50-80] | 16.0 [6.0-36.0] ^1^ | 8 [5-12] | 10 [6-15] |
|  | STR^2^ > 1.0 | 73 | 5.3 [4.5-6.0] | 61 [44-75] | 20.0 [9.0-43.0] ^1^ | 10 [7-15] | 4 [2-8] |
|  |  |  |  |  |  |  |  |
| Lee et al. (2018) | NA | 84 | NA | 5.3 (SD: 1.8) | NA | 5.5 (SD: 5.1) | 11.4 (SD: 9.2) |
|  |  |  |  |  |  |  |  |
| McWilliams et al. (2018) | ‘PPT Study’ | 77 | 4.7 (SD: 0.9) | 53 (SD: 23) | 21 (SD: 13) | 1 (SD: 1) | 11 (SD: 7) |

^1^ = CRP (mg/dl) instead of ESR; ^2^ = STR means Swollen to Tender joint count Ratio
